# Baculovirus immediately early 1, a mediator for homologous regions enhancer function *in trans*

**DOI:** 10.1186/1743-422X-7-32

**Published:** 2010-02-10

**Authors:** Xu'ai Lin, Yin Chen, Yongzhu Yi, Zhifang Zhang

**Affiliations:** 1 Department of Medical Microbiology and Parasitology, School of Medicine, Zhejiang University, Hangzhou 310058, China; 2 Biotechnology Research Institute, Chinese Academy of Agricultural Sciences, Beijing 100081, China

## Abstract

**Abstract:**

**Background:**

Enhancers are DNA sequences that serve as binding sites for regulatory proteins, and stimulate transcriptional activity independent of their positions and orientations with respect to the transcriptional initiation site. Previous studies considered that baculovirus *homologous regions (hrs)* function as enhancers in cis. In our study, a plasmid containing homologous region 3 *(hr3)* enhancer from *Bombyx mori nucleopolyhedrovirus (BmNPV) *failed to enhance transcription of promoter in other plasmid in co-transfection assays, but strong stimulation occurred when cells were infected by BmNPV.

**Results:**

The cotransfection results of each BmNPV genomic library plasmid, *hr3* plasmid and reporter plasmid showed that there were eight library plasmids stimulated the luciferase gene expression remarkably. Sequencing these plasmids revealed that each of them contained the *ie-1* gene. Transfected plasmids, containing *ie-1*, *hr3* and various origin promoter drove reporter gene showed the function was even retained. Cotransfection of *hr3* functional dissected fragment and* ie-1* revealed that the 30-bp imperfect palindrome destroyed fragment can't enhance reporter gene expression even though transfected with *ie-1*.

**Conclusion:**

IE-1 was the only early factor of BmNPV that could act as a mediator for hr enhancer function in trans and the trans-function was achieved with a broad-spectrum of promoters. The 30-bp imperfect palindrome was the elementary molecular structure by which IE-1 participated in the enhancer function in trans.

## Background

The genome of baculovirus contains interspersed *homologous regions *(*hrs*) that function as transcriptional enhancers linking *in cis *to viral or heterologous promoters in either insect or mammalian cells [1]. The immediately early gene 1, *ie-1*, is one of six essential genes required for DNA replication in transient replication assays, and the 67-kDa encoded product of *ie-1 *is the principal transcriptional regulator of baculovirus [2]. As assayed by plasmid transfection, IE-1 transactivates the expression of various baculovirus early genes and some housekeeping genes [3]. When the affected promoter links *in cis *to the *hr *enhancer, IE-1 protein also markedly stimulates promoter activity through binding to the 28-mer palindrome units [4-6].

Transcriptional enhancers for eukaryotic genes are binding sites for regulatory proteins; they lie at a distance upstream or downstream of the transcriptional start sites, and the regulatory proteins that bind to them activate (or sometimes inhibit) transcription [7,8]. A previous report showed that the *hr *enhancer stimulated transcription only in the *cis*-linked conformation [9]. In contrast, another study found that when plasmid p39CAT was co-transfected with *Bgl*II-digested viral DNA and a *Pst*I DNA library of *Autographa californica *Nucleopolyhedrovirus (AcNPV), the CAT activity increased remarkably [10].

In our study, *hr3 *from BmNPV failed to enhance the expression of the *luciferase *gene (*luc*) *in trans *in co-transfection assays, but strong enhancement occurred when the two independent plasmids were co-transfected into silkworm cells along with BmNPV. Therefore, we assumed that certain viral factor(s) participate in the trans-activation effect. A random BmNPV genomic library was constructed and used to screen viral factor(s) mediating *hr3 *enhancer function *in trans *through co-transfection with DNAs from reporter plasmid and *hr3 *enhancer-containing plasmid. According to the structural characteristics of the *hr3 *enhancer, dissection analyses with different amounts of palindromes were conducted to uncover the basic requirement for *hr3 *enhancer function *in trans*.

## Methods

### Materials

T4 DNA ligase, platinum *pfx *DNA polymerase and the lipofectin kit were purchased from Invitrogen (USA). *Taq *DNA polymerase, restriction endonucleases, pGEM-T easy vector, DNA purification kit, luciferase assay kit and pRL-CMV vector for internal control transfections were purchased from Promega Corp (USA). *E. coli *strain DH10B was maintained in our lab. The reporter plasmids pKS-hel510-luc, pKS-Bmgp64-luc and pGEM3Z-lsp-luc, containing *helicase*, *gp64 *and the silkworm *larvae serum protein *(*lsp*) gene promoter respectively, were from our previous work [11-13]. The enhancer vectors, pKS-hr114, pKS-hr198 and pKS-hr3 containing 0, 1 or 3 30-bp incomplete palindromes respectively, were constructed and maintained in our lab [14].

### Virus, cell lines and random library

The BmNPV-ZJ8 strain was maintained in our lab. *Bm*-N cells were propagated at 27°C in TC-100 insect medium supplemented with 10% heat-inactivated (56°C, 30 min) fetal bovine serum (FBS) (Invitrogen). The details for cell culture were from Summers and Smith's manual [15]. A random genomic library of BmNPV was constructed according to the "partial filling-in" method that contained a 3 kb to 5 kb fragment in the pUC19 vector [16,17]. Plasmid DNAs of 238 positive colonies were extracted for further transient assays [17].

### Transfection in insect cells

*Bm*-N cells were seeded in 24-well plates and allowed to attach at 27°C overnight. Transfection assays were conducted using lipofectin following the manufacturer's instructions. The co-transfection solution contained 0.3 μg reporter plasmid DNA, 0.1 μg internal control plasmid DNA in some cases, 0.3 μg of each plasmid DNA from the random library, and *hr *enhancer when necessary, along with 2 μl lipofectin in a total volume of 50 μl. pBlueScript DNA was introduced in some reactions to maintain a constant quantity of DNA. If virus infection was required, the virus was added to the serum-free medium and left for 1 h before the supernatant was replaced with complete medium. Each transfection contained at least three separate experiments.

### Luciferase activity assay

The cells were harvested at 48 h post transfection (hpt) and cell extracts were prepared following the instructions with the luciferase assay kit (Promega). The amount of protein in the lysate was measured using the Bradford method [18]. Measurements of dual-luciferase activity were performed with a liquid scintillation spectrometer (Beckman LS6000 Series, USA) [19]. Luciferase activity was indicated as counts per minute (CPM) in 15 s.

### Cloning of *Orf121*, *Orf122 *and *ie-1 *genes

Using BmNPV ZJ-8 DNA as template, the intact ORFs and corresponding 5' untranslated region (5'-UTR) were amplified. Primers (Table 1) were designed according to the sequence of BmNPV T3 strain (GenBank accession no. L33180). The amplified fragments were subsequently cloned into the pGEM-T easy vector, and were confirmed by direct sequencing.

**Table 1:** Primers used to amplify the *orf121*, *orf122 *and *ie-1 *genes, as well as 5' UTR

Genes	Primer name	Sequences (5'-3')
*orf121*	Orf121-F	CACGACGCGCAGCGATGATTAC
	Orf121-R	GAAAGCCACTCTTCATAATAACAAG
*orf122*	Orf122-F	CAGTGGTCTTGAGCAAACATTCC
	Orf122-R	CTTGTTATTATGAAGAGTGGCTTTC
*ie-1*	Ie1-F	GCACAGACAAAATGTGCCACACTTG
	Ie1-R	CCAACTCCCATTGTT ATTATGCAAC

## Results

### Function of the hr enhancer *in trans *via virus infection

The *helicase *promoter of BmNPV was rather weak in transient assays, and only just-detectable luciferase activity was found. When the transfected cells were infected with BmNPV, or co-transfected with reporter plasmid and pKS-hr3, transcription of *helicase *promoter was slightly augmented, that is to say, *hr3 *did not appear to function as an enhancer when presented in separate plasmids in insect cells. However, if the co-transfected cells were infected by BmNPV, luciferase activity was markedly increased, by 58447.7-fold. This result suggested that the *hr *enhancer stimulated the individual promoter when viral factor(s) were present even when they were presented in separate plasmids. A similar result (28454.5-fold) was obtained using the *lsp *promoter, a eukaryotic promoter from the silkworm larva (Table 2). We assumed that certain viral factor(s), which served as mediator(s) for the hr enhancer, functioned *in trans *to greatly stimulate transcription.

**Table 2:** Transactivation effects of *hr3 *enhancer on target promoters *via *BmNPV infection in *Bm*-N cells

Reporter plasmids	pSK-hr3	BmNPV	CPM	Fold-stimulation
pKS-hel510-luc	-	-	22.3 ± 4.5	1.0
	+	-	17 ± 3.8	0.8
	-	+	80 ± 13.3	3.6
	+	+	1303384 ± 74692	58447.7
pGEM3Z-lsp-luc	-	-	8 ± 2.7	1.0
	+	-	11.6 ± 4.1	1.5
	-	+	68 ± 21.2	8.5
	+	+	227636 ± 37514	28454.5

Luciferase activity indicated as CPM in 15 sec (minus background counts of cells). Transactivation of *hr3 *enhancer with or without virus infection presented as stimulating fold over each corresponding reporter plasmid transfection alone, that was arbitrarily set as 1.0. Each reaction contained 10 μg of protein extracted from the transfected cells.

### Genome-wide screening for viral factors mediating the hr enhancer function *in trans*

Since the *hr *enhancer functioned *in trans *in the presence of viral factors, a random genomic library was constructed for high-throughput, genome-wide screening of viral factors. The sreening was carried out by co-transfection of reporter plasmid and *hr *enhancer, along with each member of the library. Eight plasmids, which greatly increased luciferase activity in transient expression, were screened from the random library. In contrast, without the *hr *enhancer, each of the eight library plasmid DNA products still slightly stimulated the transcription of *helicase *promoter. This result suggested that each of these library plasmid DNAs contained a transactivator-coding region. Other 230-library plasmid DNAs did not stimulate *helicase *transcription whether *hr *enhancer was present or not. After sequencing, the corresponding sequences inserted in the eight plasmids were aligned with the BmNPV T3 strain genome sequence, and the intact ORFs in each plasmid are listed in Table 3.

**Table 3:** Plasmids involved in *hr3 *enhancer function *in trans*

Plasmid No.	Corresponding site in T3 strain	Intact coding regions contained	CPM
19	114443-119152	odv-e18, odv-ec27, orf-121, orf-122, ie-1	202023
58	115898-119619	orf-121, orf-122, ie-1	554392
186	115898-119152	orf-121, orf-122, ie-1	100268
262	115216-119619	orf-121, orf-122, ie-1	112732
280	114846-120318	odv-ec27, orf-121, orf-122, ie-1, odv-e56, orf-125	381711
289	115216-119619	orf-121, orf-122, ie-1	261497
310	114846-120549	odv-ec27, orf-121, orf-122, ie-1, odv-e56, orf-125	159852
347	115898-119152	orf-121, orf-122, ie-1	717312

All the coding regions were presumed according to the complete genome sequence of the BmNPV T3 strain (GenBank accession no. L33180). Luciferase activity indicated as CPM in 15 sec (minus the background counts of cells). Each reaction contained 10 μg protein extracted from the transfected cells. The CPM values obtained from the transfection of *helicase *promoter containing reporter plasmid alone were in the range of 18 to 40.

### IE-1 protein affected hr enhancer function *in trans *alone

According to the screened regions, *odv-e18, odv-ec27, odv-e56 *and *orf-125 *were included in some of the eight plasmids, so we considered that these genes were not concerned with the enhancement of promoter activity, while another three genes, *orf-121, orf-122 *and *ie-1*, were all included in each of the 8 plasmids. Based on the complete genomic sequence of the BmNPV T3 strain, ORF-121 and ORF-122 are hypothetical proteins encoded by an intergenic region between IE-0 and IE-1, with molecular weights of 11 and 23 kDa respectively. To investigate whether ORF-121, ORF-122 or IE-1 protein alone is sufficient to recover the ability of *hr *enhancer to function *in trans*, *orf-121*, *orf-122 *and *ie-1 *with their promoter regions were cloned into pGEM-T easy vector, respectively. The recombinant plasmid was used for co-transfection assays to identify whether these three gene products participated in *hr *enhancer function *in trans*. Two BmNPV-derived promoters, *helicase *and *gp64*, host-derived *lsp *promoter and mammalian virus-derived *CMV *promoter/enhancer regions were used for the test. All the results revealed a stimulatory effect ranged from 40 to more than 100 folds as shown in table 4 when co-transfected reporter plasmid, hr3 plasmid and ie-1 plasmid. It demonstrated that the trans-function of enhancer on a broad-spectrum of promoters was achieved through the involvement of IE-1 protein while not through orf121 or orf122 protein.

**Table 4:** Transactivation effects of *hr3 *enhancer on target promoters *via *IE-1 protein bridge.

Reporter plasmids	pGEM-T-ie1	pSK-hr3	CPM	Fold-stimulation
pKS-hel510-luc	-	-	21.4 ± 5.6	1.0
	+	-	16093 ± 1432	752
	+	+	735880.1 ± 119032	34387
pSK-Bmgp64-luc	-	-	74.9 ± 15.4	1.0
	+	-	40080 ± 2947	535
	+	+	5738241 ± 609222	76612
pGEM3Z-lsp-luc	-	-	14.4 ± 3.9	1.0
	+	-	208 ± 17.3	14.4
	+	+	26876 ± 4811	1866
pRL-CMV	-	-	78 ± 11.8	1.0
	+	-	404 ± 53.5	5.2
	+	+	76400 ± 9312	979.5

Luciferase activity indicated as CPM in 15 sec (minus the background counts of cells). Transactivation of hr3 enhancer with or without *ie-1 *gene (0.1 μg DNA in each transfection) presented as stimulating fold over each corresponding reporter plasmid transfection alone, which was arbitrarily set as 1.0. Each reaction contained 10 μg of protein extracted from the transfected cells.

### Functional dissection of hr enhancer structure essential for function *in trans*

**Figure 1:**
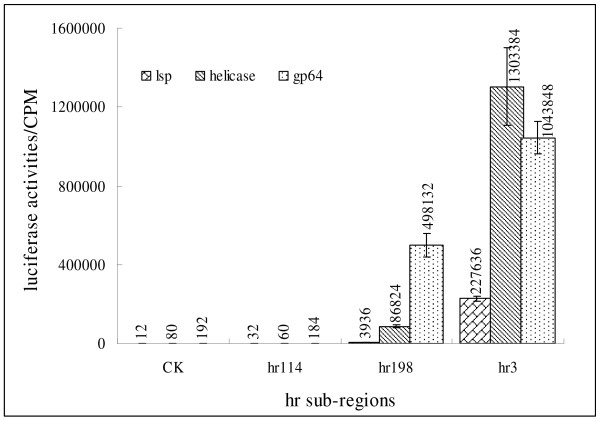
**Transactivation effects of *hr3 *enhancer sub-regions on different promoters *via *virus infection**. Reporter plasmids containing different promoters were co-transfected with the *hr *derivates by virus infection. 10 μg of protein was extracted from the transfected cells to determine the luciferase activity. hr114 is a plasmid containing a fragment without an intact 30-bp incomplete palindrome but half of the palindrome on both sides; hr198 contains one 30-bp incomplete palindrome; hr3 contains an intact 651-bp *hr3 *fragment with 3 palindromes. CK had no hr added.

**Figure 2:**
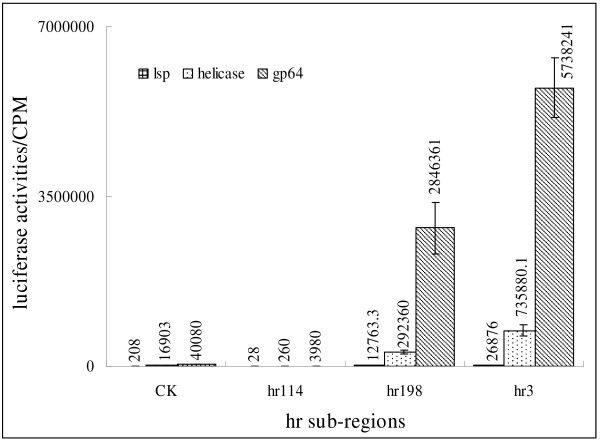
**Transactivation effects of *hr3 *enhancer sub-regions on different promoters *via *co-transfection with pGEM-T-ie1 DNA**. Reporter plasmids containing different promoter were co-transfected with pGEM-T-ie1 and *hr *derivates. 0.1 μg of pGEM-T-ie1 plasmid DNA was used in each transfection. Each luciferase reaction contained 10 μg of protein extracted from the transfected cells. CK, no hr added; hr114, plasmid containing a fragment without intact 30-bp incomplete palindrome but half of the palindrome on both sides; hr198 contained one 30-bp incomplete palindrome; hr3 contained an intact 651-bp *hr3 *fragment with 3 palindromes.

The 651-bp *hr3 *enhancer of BmNPV contains three direct repetitive regions, each of which contains a 30-bp incomplete palindrome with a naturally occurring *Eco*RI site as the core. The plasmid pKS-hr114 contains a 114 bp enhancer fragment from *hr3 *with no intact 30-bp incomplete palindrome but with half of the palindrome on both sides. pKS-hr198 contains one 30-bp incomplete palindrome, and pSK-hr3 contains an intact 651-bp *hr3 *fragment with 3 palindromes. The incomplete palindrome is the vital structure for *hr *enhancer function *in cis*. To evaluate the effect of the palindrome on enhancer function *in trans*, and further understand how ie-1 participates in the enhancer function *in trans*, reporter plasmids were co-transfected with the *hr *derivates, by virus infection or cotransfected with the *hr *derivates and pGEM-T-ie1. Results revealed that the palindrome was essential to *hr *enhancer function *in trans*. pKS-hr114 did not stimulate transcription from affected promoters even by virus infection or co-transfection with pGEM-T-ie1. The constructs with intact palindrome(s) dramatically increased the transcription of the reporter gene in the presence of IE-1 protein (Fig. 1 and 2).

## Discussion

Baculovirus homologous regions are repeated sequences that are interspersed in the genomes of baculoviruses. It is known that these regions contain the origins of DNA replication, and augment the expression of a number of genes in an orientation-independent manner [9].

In the random genomic library of BmNPV, the average size of fragments were 3 to 5 kb, while the largest open reading frame of BmNPV, *helicase*, was 3669 bp, so we ensured that the largest coding region in the genome was included in the random library. According to the average size of cloned fragments, the representation of the random library was more than 99% [20]. Therefore, the completeness and representation of the constructed library was adequate for genome-wide screening for regulatory products or elements.

In some cases, enhancers can function *in trans *on a separate DNA molecule *via *a protein bridge to the promoter by covalent linkage of molecules or a cellular factor binding with the two elements [21,22]. The simian virus 40 (SV40) enhancer functions *in trans *to the β-globin promoter when they are linked by a protein bridge [21]. A recent report even suggests that certain enhancers can interact with several target promoters *in trans *on different chromosomes [23]. Genome-wide screening revealed eight plasmids, all containing *orf-121*, *orf-122 *and *ie-1 *coding regions could stimulate the expression of luciferase. Subsequent experiments showed that IE-1 was the only factor of BmNPV that acted as a mediator for the *hr *enhancer functioning *in trans*. This may be because the sizes of the two hypothetical proteins ORF121 and ORF122 are too small to afford the binding domain and activating domain required for a transcription factor. Previous reports using gel shift assays and mutational analyses confirm that IE-1 binds directly to the *hr *enhancers [24,25]. The direct interaction between IE-1 and *hr *enhancer may be consistent with the theory that some eukaryotic enhancers reach their targets (as yet undefined) by means of DNA loops, IE-1 bring the *hr *enhancer to the proximal promoter and the complex is easily recruited by the transcriptional apparatus [26,27]. In this study, we assumed that IE-1 plays two distinct roles, one as a transactivator to stimulate the transcription of the basal promoter, and the other as a mediator for *hr *enhancer functioning *in trans *by binding to the hr enhancer and then reaching the target promoter to give another 40 to 100-fold activation. So the enormous enhancement of luciferase activity was obtained by the cumulative effects of these mechanisms. When hr114 was co-transfected with pGEM-T-ie1 and reporter plasmids, the CPM values were lower than control assays with co-transfected pGEM-T-ie1 and reporter plasmids. Since IE-1 can bind with a half palindrome of *hr *enhancer, the competitive binding of IE-1 by the two half palindromes might have resulted in the decrease of IE-1 transactivator. It was reported that the imperfect palindrome, especially the naturally occurring *Eco*RI site, is essential for its enhancing function *in cis *[25]. The present study demonstrated that the intact palindrome is also the elementary structural requirement for *hr *enhancer functioning *in trans*.

AcMNPV *hr5 *functions *in trans *in an IE-1-dependent *39K *promoter and the *p35 *promoter, and the stimulating effects of *hr5 in trans *are about 2 and 7-fold [28,29]. In this report, the IE-1 induced much higher activation of all the differently derived promoters to the *trans*-presented *hr3 *enhancer from BmNPV, and this suggests that IE-1 is a generic mediator for *hr *enhancer functioning *in trans*. Screening of the genomic library confirmed that IE-1 is the only viral factor that mediates *hr *enhancer functioning *in trans*. Furthermore, this result implies that in some transfection experiments, particularly for co-transfection molecules and internal controls, the trans-effects should not be ignored [30].

## Competing interests

The authors declare that they have no competing interests.

## Authors' contributions

XAL and YC performed the experimental work and analyzed the data. YZY contributed to the cell culture. XAL, YC and ZZF conceived the experimental strategies and designed the experiments. All authors read and approved the final manuscript.
